# A digital workflow for modeling of custom dental implants

**DOI:** 10.1186/s41205-019-0046-y

**Published:** 2019-06-06

**Authors:** Andrejus Surovas

**Affiliations:** Private practice, “Dantu Implantacijos Klinika”, Kalnieciu 100, LT50184 Kaunas, Lithuania

**Keywords:** Subperiosteal implant, Custom dental implant, Advanced bone resorption, Medical device modeling, Implant modeling, Modeling software

## Abstract

Modern dental treatment with standard screw-type implants leave some cases unaddressed in patients with extreme jaw bone resorption. Custom-made subperiosteal dental implant could be an alternative treatment modality to sinus lift, nerve lateralization or zygomatic implant techniques. Subperiosteal dental implants were utilized for many years to treat such patients. A combination of traditional subperiosteal implant designs with current advancements in 3D imaging, design and printing allow to reduces treatment time and provides abutments for prostheses in cases where other techniques do not provide satisfactory results. The data manipulation and design software are important aspects in the manufacturing of custom implants. Programs that are specialized for industrial or medical design typically cost tens of thousands of US dollars. In this work I establish and test steps for design and production of a custom medical device (subperiosteal implant) from patient computed tomography (CT) data. Work stages to be defined are: selection of necessary software, CT data processing, 3D virtual model creation, modeling technique for custom implant and data file preparation for printing. Patient CT data was successfully converted into a watertight STL (Standard Tessellation Language) model of the maxilla. Error corrections and design were completed using freely available programs from Autodesk Inc.. The implant was produced in Ti64 (a type 5 titanium alloy) using three-dimensional (3D) printing DMLS (direct metal laser sintering) process. The avoidance of high cost software makes this treatment modality more accessible to smaller clinics or mid-size production facilities and subsequently more available to patients.

## Introduction

As populations age, the need for dental treatment of partially or fully edentulous patients increases. Nowadays, seniors often are socially and physically active and expect a high quality of life. One important aspect of such quality of life is healthy dentition, or at least non-removable teeth prostheses. Dental treatment plans often include implant placement, which in turn requires sufficient bone quantity. However, elderly people usually do not have the amount of bone required for standard root form implant placement. Moreover, elderly patients are poor candidates for bone augmentation due to their decreased metabolic rate and decreased regenerative capacity [1]. When bone augmentation is not possible, implant placement is no longer an option.

Thus, dental professionals are faced with demands for different solutions to restore dentition with non-removable teeth prostheses.

Subperiosteal dental implantation, a conservative option for implant treatment of severely resorbed maxilla and mandible, has existed for quite a long time. It was first introduced by doctor Gustav Dhal in 1937 in Sweden [2]. Nowadays, with advances in regenerative medicine, this option has all but been forgotten by the dental community, even though it could be an effective and useful option in the treatment of patients with heavily resorbed jaws.Conventionally, this implant is produced in two stages (involving two surgeries). During the first surgery, an impression is taken of the jaw bone. Then, a custom subperiosteal implant is made in the laboratory and placed on the jaw bone in a second operation. However, patients are not very receptive of this kind of two-stage surgery for elective treatments.

With the advent of computed tomography (CT) and freeform fabrication techniques (such as stereo-lithography and fused deposition modeling), new approaches for patient treatment were introduced in medicine in general, and in dentistry in particular. Rapid prototyping technology expanded the use of CT beyond diagnostics into surgical planning and the making of patient-specific tools and implants. It thus became possible to make anatomical models and patient-specific implants for different anatomic locations (such as skull parts, vertebrae, and hip joint replacement components) and patient-specific instruments (e.g., surgical guides) [3–6]. Attempts to produce custom orthopedic prostheses based on CT imaging data have been reported since 1985. The first documented case of an orthopedic device made using 3D digital technology was a mandibular subperiosteal implant made by James RA [7]. At that time, direct metal printing was not available; therefore, CT data were used to create anatomical models of patients’ mandibles, which were then actualized as implants using traditional metal casting technology.

After direc metal laser sintering (DMLS) technology became available from EOS (Krailling, Germany) in 1995 [6], it became possible to make three-dimensional objects in metal with virtually any complexity. This opened up possibilities for completely digital manufacturing processes—a route from patient anatomy virtualization to digital design and direct printing in biologically compatible metal or alloy (such as steel, CoCr (cobalt-chromium) alloy, Ti64 alloy, or pure titanium). Introduced into the dental field, these techniques expanded the ways in which custom subperiosteal implants could be made. With currently available technologies (as of 2018), it is possible to make subperiosteal dental implants using any of the following four methods:

1. Conventional: bone impression and lost wax casting metal
2. Transitional: CT, 3D jaw model printing, lost wax casting in metal
3. Digital: CT, modeling on virtual jaw model, printing in metal using DMLS or similar technology
4. Hybrid: bone impression, silicone impression or stone model optical scanning, modeling of implant on virtual jaw model, printing in metal using DMLS or similar technology

The first method is the known classic, long-established approach that requires a second surgery for implant placement. During the first surgery, an impression is taken with silicone material. Then, the implant is modeled on a refractory model, cast in metal, and finished between surgeries—usually 2–3 weeks. The implant is put into place in a second surgical procedure.

The second approach avoids the second surgery by using CT data obtained in advance and a refractory model for casting, duplicated from a 3D printed model. This method assumes that the user has access to CT and 3D freeform manufacturing in plastics, but has no access to metal printing technology. The drawback of this method is that more steps are involved than in the conventional method. Consequently, it may not be as precise (because of 3D printing and duplication steps).

The third method gives us the shortest path to the final product—custom-made implants— because no extra 3D printing or model duplication steps are involved. Most of the manufacturing takes place in the digital environment. CT data are used to make a virtual model, and the modeled implant is directly printed in the material of choice.

The fourth method appears to be unnecessary and redundant, but it has a place in practice, because the implants manufactured by the digital method do not always fit sufficiently well to be useful in practice. Once a surgeon encounters a poorly fitted implant made by the third method, he/she must follow Method 4—take an impression and postpone implant placement until a suitable implant has been made by the “hybrid” method. The use of this method might be necessary when a surgeon observes large discrepancies between the implant and bone.

This article will describe a digital approach for manufacturing of subperiosteal dental implants, conducted with the following.

## Objectives

1. Define a digital workflow from CT scan to printed implant
2. Elucidate variables affecting the quality of the end product—i.e., the implant
3. Determine the possibility of completing this task using freely available software

## Methods

### Data and software

CT data were obtained from a patient scanned at the Radiology Department of the Hospital of Lithuanian University of Health Sciences. The patient was scanned with a Toshiba Aquilion One multi-slice spiral tomograph with preset protocol (0.25 mm step, 0.5 mm slice thickness, gantry angle, 0°.). Axial slices were exported using FC30 convolution kernel. The data were anonymized using DICOM Anonymizer by Sha He (2008) [8]. The computer used was a PC with an Intel i5–3570 3.40 GHz CPU, 16 GB RAM, NVIDIA GeForce GT640 1 GB RAM video card, standard optical mouse, and running 64-bit Windows 10 operating system. The programs used for digital work are listed in Table 1.

**Table 1:** List of computer programs used in this work

Software name and version	Producer website	License type	Tasks software was used for
Sante DICOM Viewer FREE, ver. 4.0.14	www.santesoft.com	free software	• CT data set review
3D Slicer, ver. 4.5	www.slicer.org	open-source software, BSD style license	• CT data import and volume rendering
			• bone tissue segmentation
			• jaw digital model production
Meshmixer, ver. 10.9	www.meshmixer.com	free, Autodesk inc.	• virtual model manipulation
			• modeling implant
			• basic model repair
			• measurements
Netfabb Private, ver. 5.2	https://www.autodesk.com/products/netfabb/overview	commercial, Netfabb GmbH	• cutting and repair of jaw models
			• repair of implant digital models
			• measurement

### Workflow description

#### CT data-set review

First the quality of the CT scan data was assessed. This consisted of checking whether the ROI (region of interest) was as required, if number and thickness of the slices were sufficient, and if there were any scanning artifacts of various nature.

**Fig. 1:**
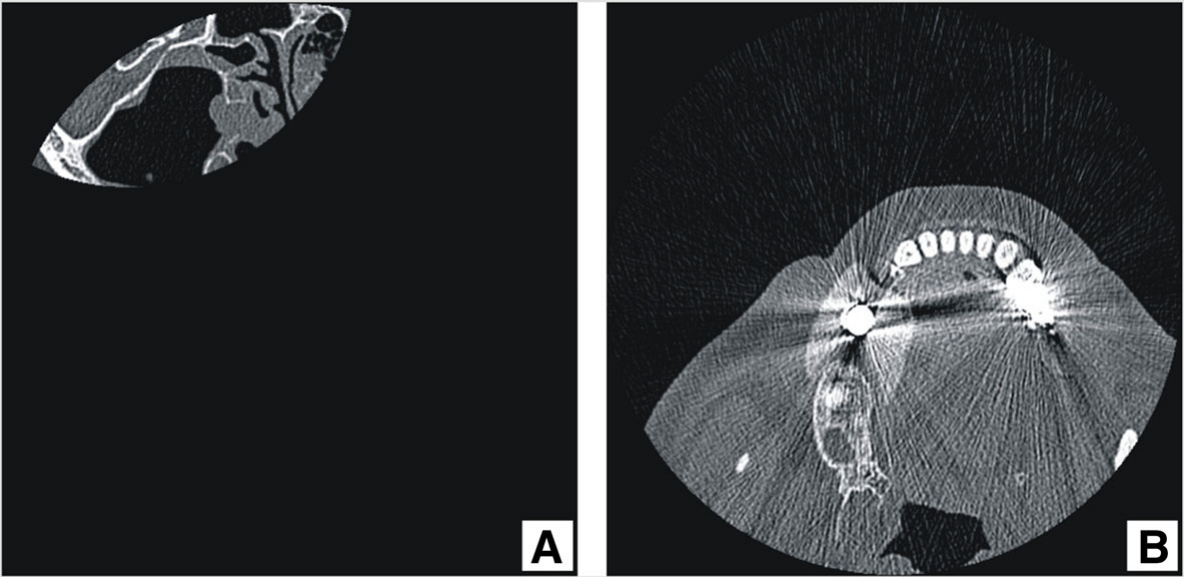
CT scan errors: **a**) incomplete image; **b**) x-ray scatter artifacts from metal objects in the patient tissues and cavities

The DICOM file header also had to be checked to determine if the parameters used during scanning were correct. The parameters include the protocol used, gantry angle, slice thickness, slice step, and convolution kernel. If the gantry angle is set greater than 0.0° and the reconstructing software is unable to make the necessary corrections, then shear distortions of the model may occur [9]. The start and the end of a CT scan images series often contains defective slices that have to be discarded on dataset review (Fig. 1a).

**Fig. 2:**
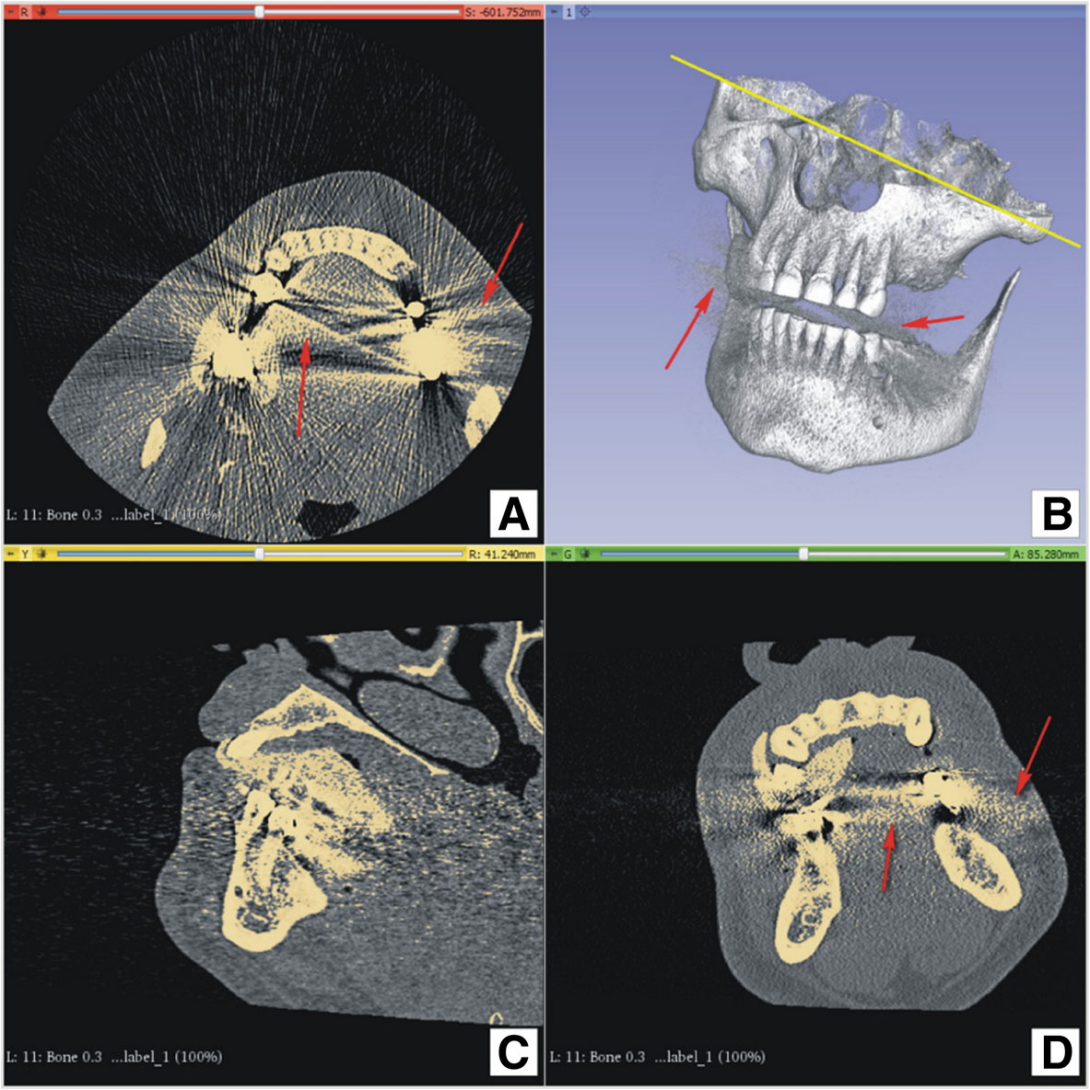
Modeling3D Slicer user interface, 4-up layout: **a**) **b**) **d**) red arrows point to x-ray scatter artifacts; **a**) yellow line emphasize angled patient positioning during CT scan, to avoid artifacts in left maxillary region, important for modeling; **a**) **c**) **d**) ivory colour depicts thresholding label which will be used for model generation

If the data obtained from the CT contains errors (e.g., metal-induced scatter or movement artifacts), it has to be determined if these errors affect the anatomical region required for future modeling (Figs. 1b, 2a, b).

If so, then a decision has to be made whether to reacquire the CT scan or to take a bone impression instead. If CT artifacts do not affect the area of the proposed implant, then one can proceed to the segmentation step. The image set also needs to be assessed for motion artifacts, which can only be seen in the reconstructed sagittal projection or 3D volume rendering.

### CT data import and virtual model production

Slicer 3D software was used to create the 3D model of the jaw. Bone tissue segmentation was performed, as explained in the online Slicer 3D tutorial [10]. The most important step is correct choice of level thresholding for the tissue of interest. The specific threshold at which the program will build a surface is influenced by many factors: CT scanner and its software settings (convolution kernel), CT volume cropping, bone density of the actual patient, and most importantly, operator selection. Human decision for threshold selection is very important and is the sum of anatomy knowledge and work experience, which has no substitute.

**Fig. 3:**
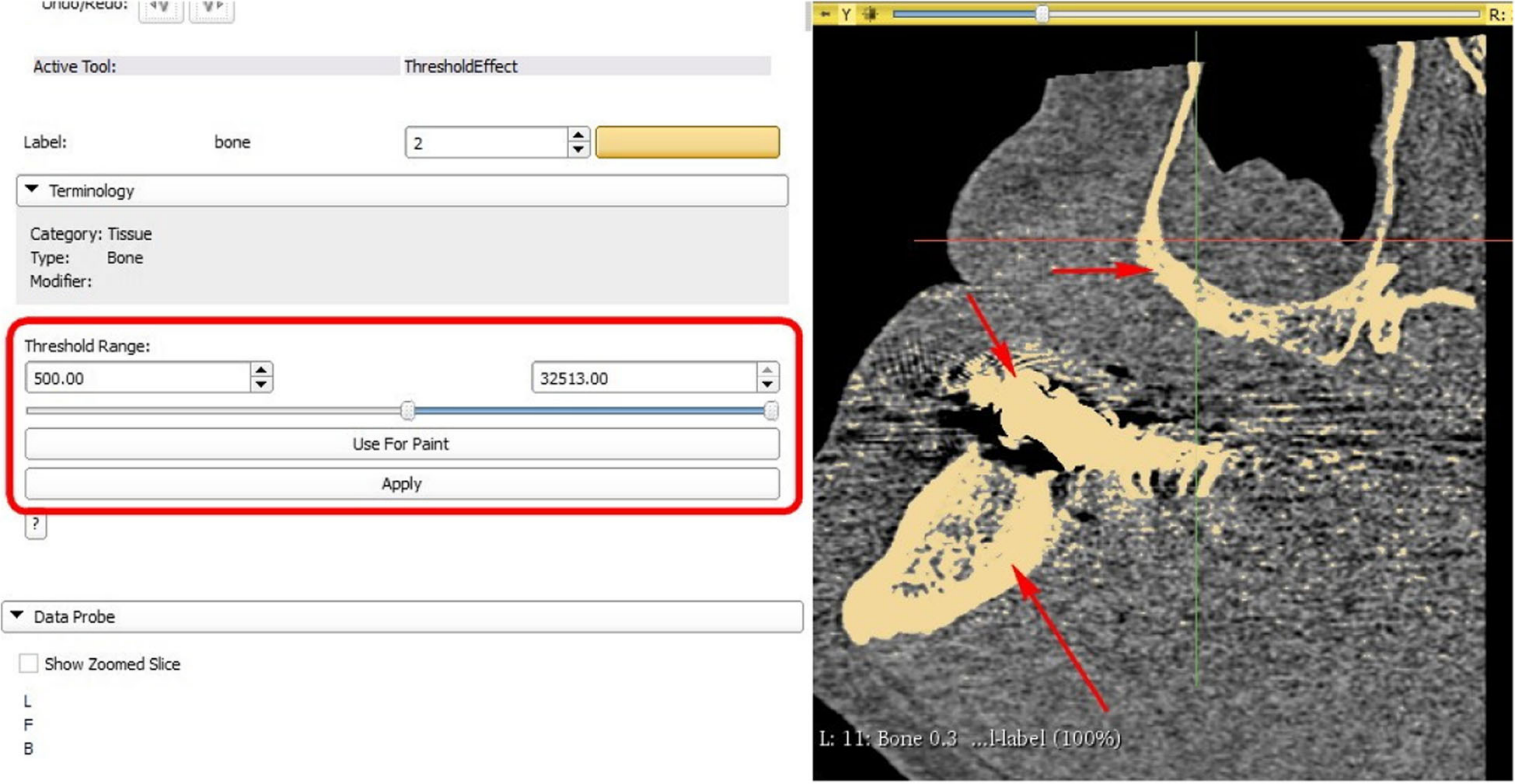
3D Slicer user interface, thresholding tool: thresholding level selected using slider or entering numerical value (depicted by red rectangle). Bone label (standard from Slicer3D) is visualized by ivory colour in slice image (depicted by red arrows)

**Fig. 4:**
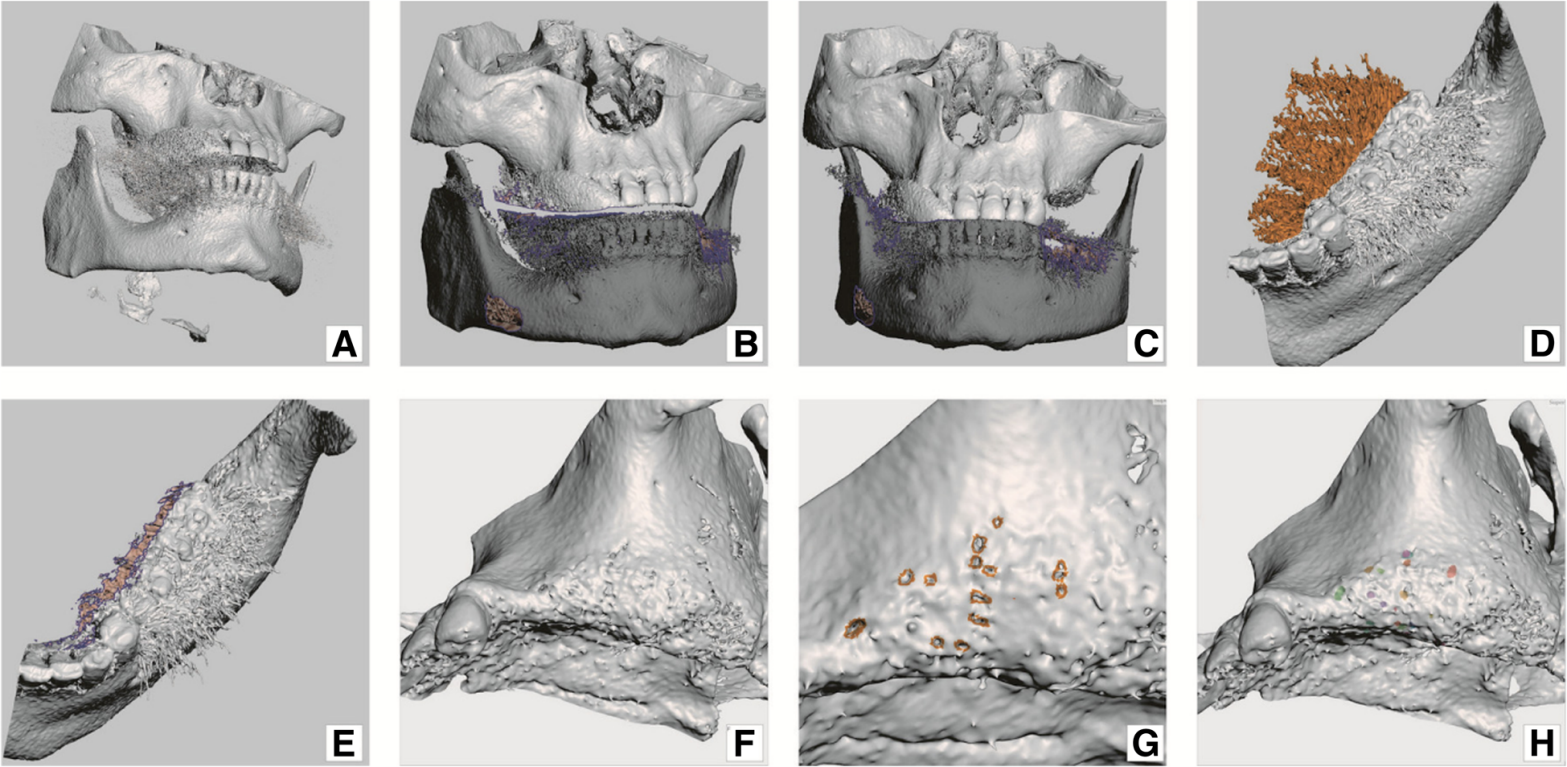
Model preparation: **a**) newly generated bone tissue 3D model (mesh); **b**) model – fragments of vertebrae, hyoid bone and some noise were removed; **c**) maxilla and mandible have put into occlusion; **d**) mandible trimmed down to size necessary for work; **e**) more noise removed from mandible; **f**) maxilla presenting anatomical holes (apertures); **g**) “healing” anatomical holes in maxilla; **h**) “healed” maxilla, colour patches represent former anatomical holes

Following selection of the desired threshold for volume label (or mask) (Fig. 3),

the surface model of the selected anatomy is created (Fig. 4a).

This is usually a polygon mesh that can be saved as a number of digital formats, such as .stl, .obj, .ply, and .vtk. In this case, .stl (STL) was used because of compatibility with all currently used programs. The STL file format describes 3D objects as meshes made of stitched triangles. Segmentation programs of all kinds, including commercial software, are prone to errors while constructing mesh models of anatomical structures. Created mesh (i.e., virtual model of the jaw) often contains non-manifold edges, holes, inverted triangles, and other errors that hinder further processing steps. Human bone with its trabecular and cavernous structures is difficult for computers to convert to manifold (closed surface) mesh structures, which is required by modeling software. This is very evident when a program tries to recreate the trabecular bone structure (Figs. 4f and g). The situation mandates the use of specialized error correcting programs such as Netfabb. Even then, the program only corrects software errors introduced by segmentation software, but not holes in the bone anatomical structure. Fenestrations in the virtual bone model may be part of the actual anatomy of the patient; they also may be artifacts of thresholding. Anatomical holes of models are to be corrected manually (holes closed) or avoided during modeling (Figs. 4g and h).

### Modeling

#### Biological considerations

The number of abutments has to be minimized to make fewer perforations of mucosa. Implant abutment in the oral environment is a very special place—it is a direct gateway for organisms from the internal environment to the external environment (oral cavity). This perforation of mucosa is the place where the organism lacks its natural barrier against microorganisms present in the oral cavity. None of the current dental implants form a firm implant-epithelial junction that can reliably prevent microorganism invasion into the bone. It has been shown than bacterial invasion leads to loosening of the implant-bone bond in the long term. This condition is even more prominent with subperiosteal dental implants of which only 30–40% of the whole surface make contact with the bone and the rest is surrounded by soft tissues. Thus, chances of bacterial invasion should be minimized by reducing the number of mucosa perforations. One must avoid creating narrow spaces, crevices, and concavities, especially near abutments. Pits and narrow spaces are harbors for possible oral fluid microorganism contamination and proliferation. In addition, crevices impede tissue liquid circulation and thus hinder regenerative processes. It is important to respect blood supply and not hinder periosteum to bone contact, such as with large metal areas. Overall, it is recommended to use as little metal as possible to achieve masticatory force distribution goals.

#### Prosthetic considerations

**Fig. 5:**
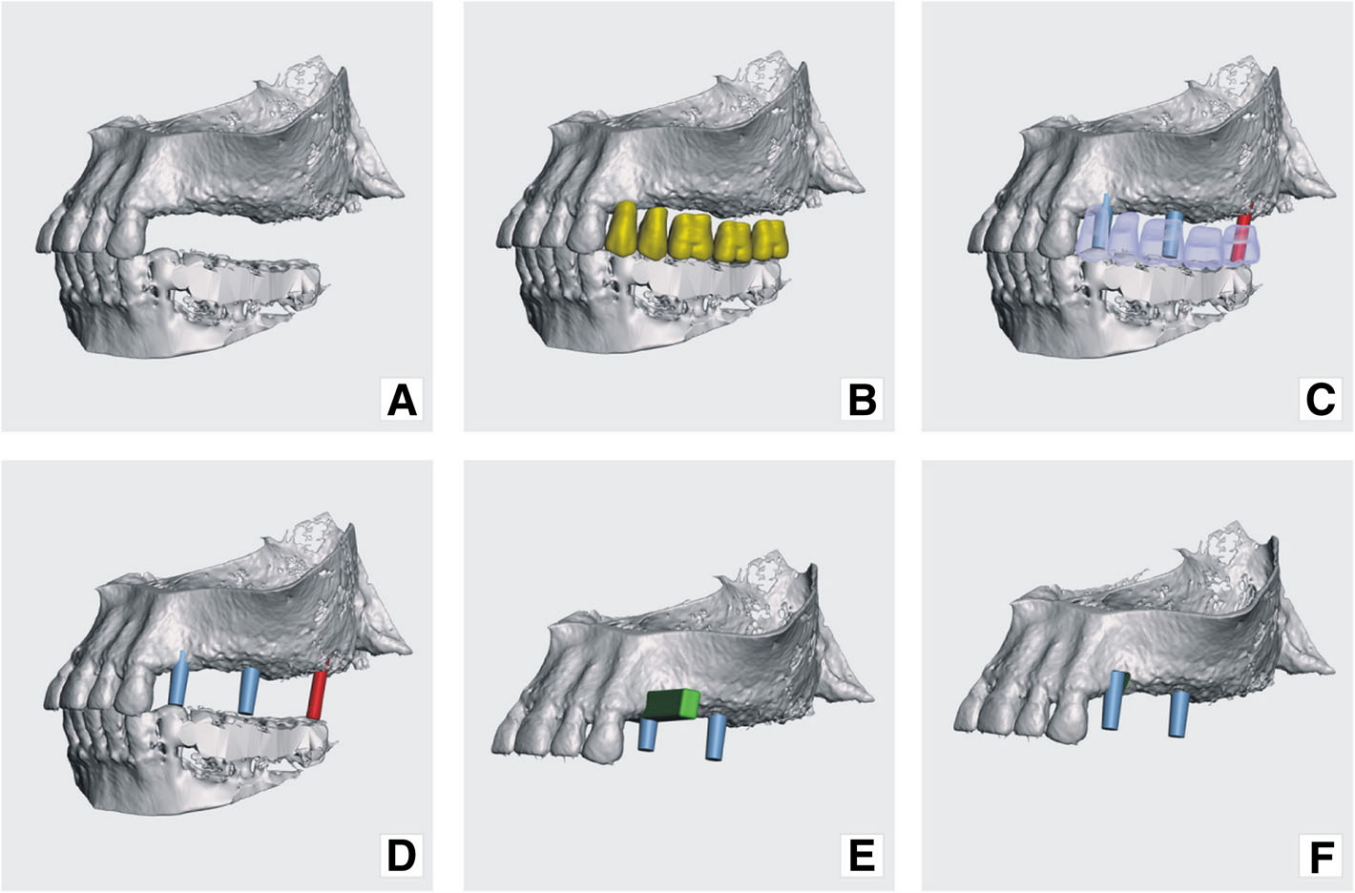
Model preparation continued: **a**) virtual jaw models in occlusion; **b**) virtual teeth added; **c**) prospective abutments have been placed in places corresponding to future teeth; **d**) future abutments – sky blue abutments belong to subperiosteal implant and red one belongs to planned screw type implant; **e**) a cut-off tool placed in the area of tooth 24 (FDI), where abutment was planned to bury deeper into the bone; **f**) maxilla with cut-off and placed abutments

One should plan dental function and aesthetic in advance, before designing the actual implant. First, virtual models have to be put into centric relation by means of existing dentition intercuspation or using x-ray markers in the removable dentures that are being used as scanning aides (Figs. 4b and c). Home work also includes examining diagnostic models and wax-ups that aid in planning abutments emergence profiles and their relation to planed prostheses. Using virtual teeth in the software during the process of implant modeling also aids proper placement of abutments (Figs. 5b and c).

In addition, consider the lateral component of jaw movements—create anatomical and mechanical means of implant fixation. When creating fixed cementable abutments, pay attention to parallelism of prospective abutments with current partnering abutments (teeth or implants) and the relation to opposing dentition (antagonists).

The modeling process of a custom dental implant per se is the de novo creation process. This is in contrast to reconstructive surgery modeling, which often uses contra-lateral mirroring to obtain correct anatomical structures. Thus, new objects of intricate shape must be created, which in turn must closely adapt to current anatomical structures. This task requires suitable digital tools. Modeling for medical applications is still a new and emerging field of digital design. There is a range of software built for the dental prosthetic field and numerous programs for implant planning and surgical guide creation. Although some of these programs offer planing osteotomies and bone grafting, the majority are predominantly limited to use of standard screw type implants from different manufacturers. Consequently, they are not useful for modeling custom structures. Numerous software packages have been created for making fixed and removable dental prostheses via CAD/CAM. It appeared that the most straightforward route would be to use applications designed for modeling a partial removable dentures because of the similarity of subperiosteal implants and the framework of partial removable denture. However, after testing several software packages, this route proved to be unusable because the programs do not allow jaw model modification, are limited to preset structures, or require heavy customization to be useful.

#### 3D modeling tools

Depending on software capabilities and designer approach, subperiosteal dental implants can be created using several different tools (techniques). The applicable techniques and corresponding programs are listed below:

° Digital clay modeling.

▪ Sculpt clay with brush tool (Meshmixer, Blender, FreeForm).

▪ Sculpt clay applying profile along surface curve (VRMesh, FreeForm, Sculpt).

° Selected surface extrusion (Meshmixer, VRMesh).

° Curve fitting with pipe or tube (VRMesh, Freeform, Sculpt).

In this case, modeling was conducted via the addition-subtraction method using the mesh brush sculpting technique. Two copies of a jaw model were used. The the first copy was used to model the implant itself by bulging virtual clay, which is similar to the manner in which a dental technician lays wax on the refractory model for partial production of a removable framework. The second copy was used for subtraction from first—to form the inner (bone facing) surface of the implant.

#### Further details

Models for the maxilla and mandible should be put as close as possible to centric relation, which makes abutment planning easier (Figs. 4b and c). Placing virtual teeth helps with the planning of future occlusion and abutments (Figs. 5b and c).

**Fig. 6:**
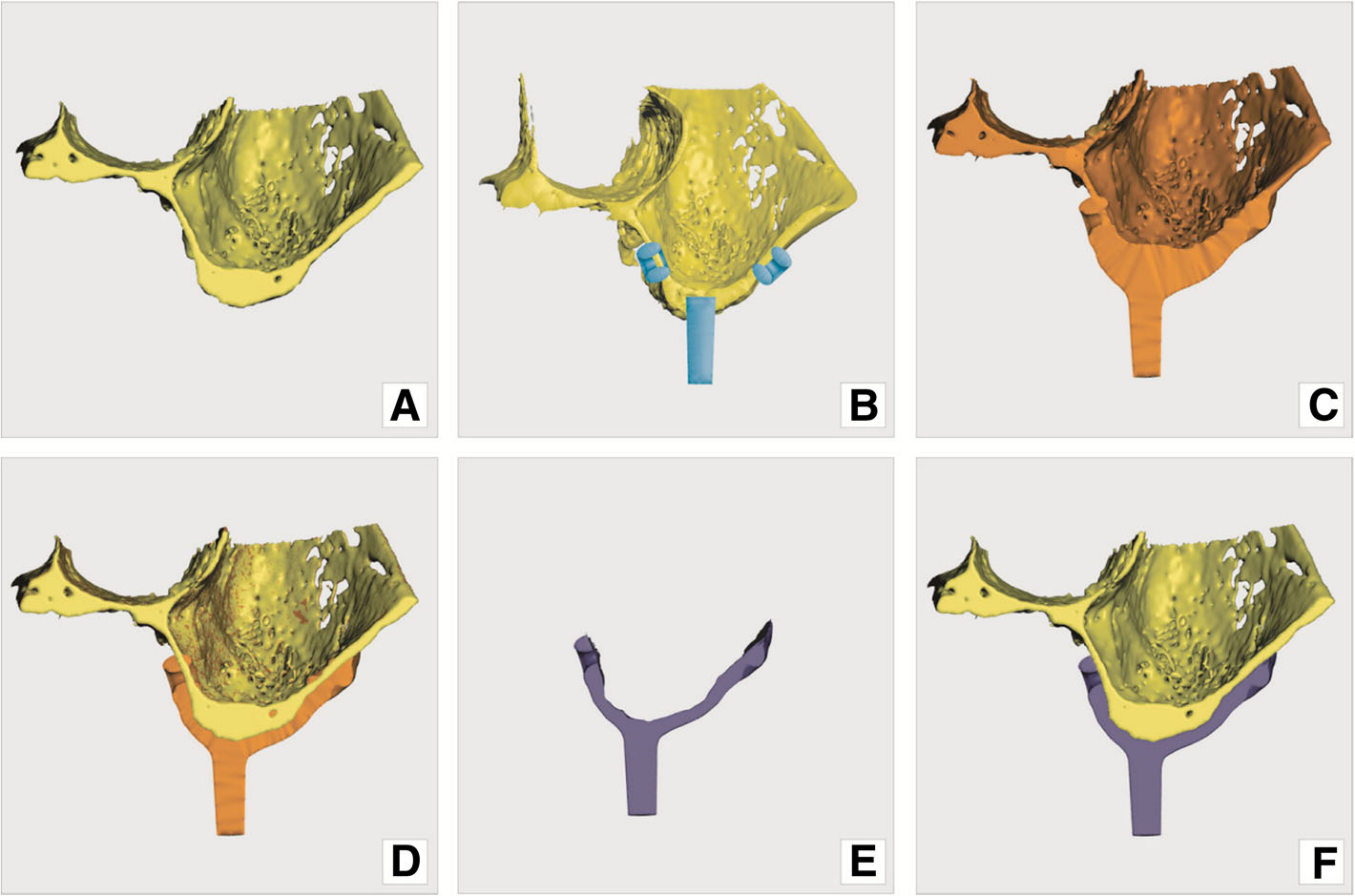
modeling principle: **a**) original model of the jaw; **b**) pre-made structures (loops and abutments) added on to virtual model; **c**) pre-made structures and modelled (bulked) implant merged with original model; **d**) the 2nd copy of original model (yellow) recalled and matched with model onto which implant was modelled (orange); **e**) implant – result of Boolean operation, subtraction of yellow model form orange model; **f**) implant (resultant) model superimposed on the original jaw model. (Screenshots from Meshmixer program)

**Fig. 7:**
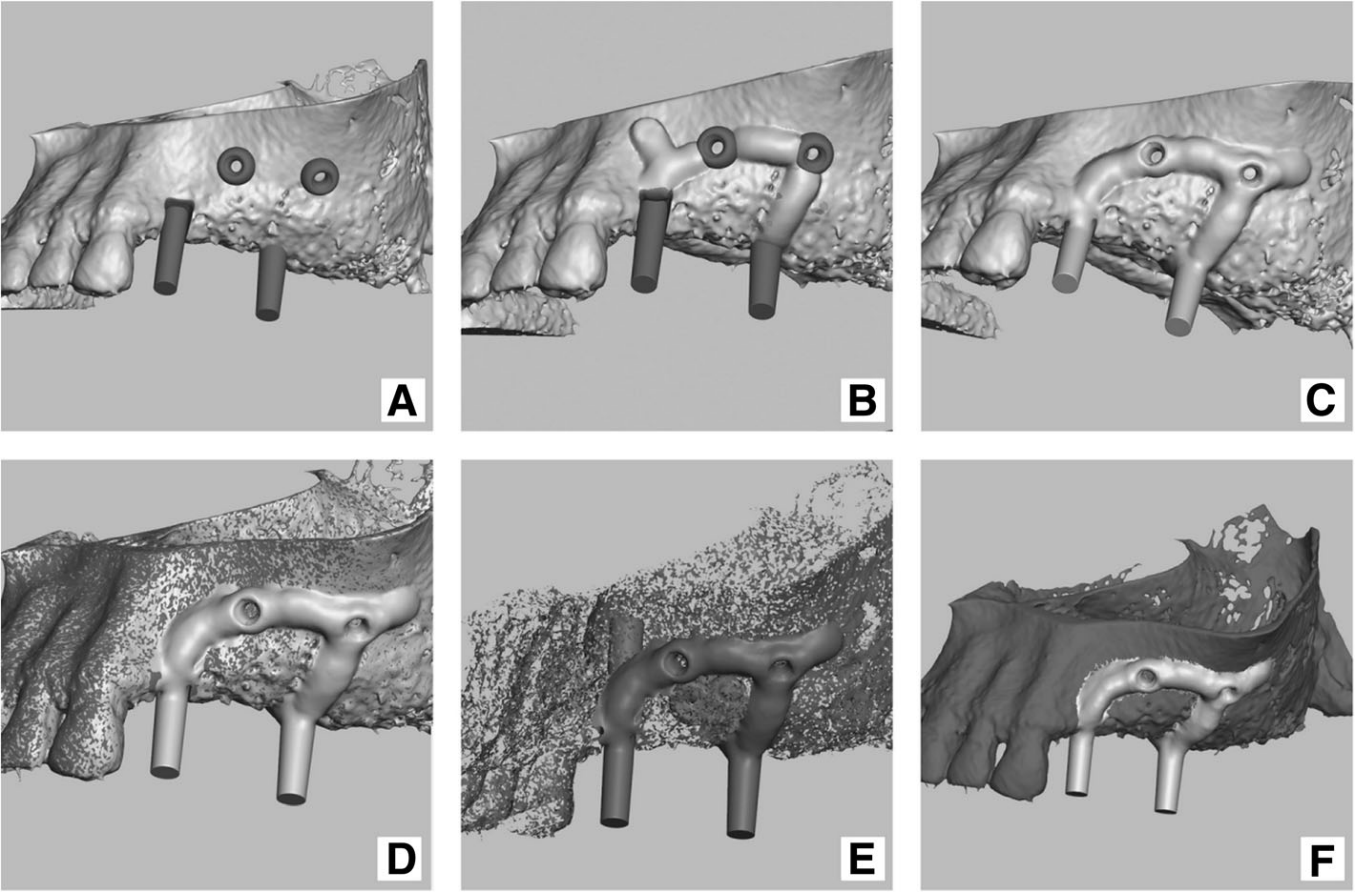
modeling in Meshmixer program: **a**) pre-made structures (loops and abutments) added on to original jaw model; **b**) implant modeling (bulking) by sculpt tool; **c**) pre-made structures and modelled (bulked) implant merged with original model; **d**) the 2nd copy of original model (dark grey) recalled and matched with model onto which implant was modelled (light grey); **e**) implant – result of Boolean operation (with lots of unwanted shells), **f**) implant (resultant) model superimposed on the original jaw model

Firstly, abutments are placed according to the current bone anatomy and future prosthetic plan. Bone quality and anatomic form must be considered while designing the implant. Load bearing and retentive elements should be planned only where at least 0.8 mm of cortical bone is present. Retentive elements (loops, puds) were placed at specified locations and all above-mentioned elements were merged with one copy of the jaw models using a Boolean addition operation (Figs. 6b and c, Figs. 7a and d).

During the third step, interconnecting structures (straps) are modeled into saddle-like web connections using various brushes of the “SCULPT” tool in Meshmixer (Figs. 7b and c). Abutments, retention loops, and peddles are connected by straps. The overall structure of the subperiosteal implant is usually designed to be 1.5–1.8 mm thick, which is an empirically defined value. During modeling, the implant is shaped slightly thicker by 0.2–0.3 mm to allow for later reduction and smoothing of its surface. Reduction is necessary because of the nature of the DMLS process, which results in printed parts with rough and oxidized surfaces. Supports also leave rough surface once removed; thus, extra thickness of metal must be included to enable reduction of the material.

#### Boolean subtraction

The Boolean subtraction operation is an essential part of custom implant additive modeling that results in a final product. The input for this computation consists of two models. The first model is an anatomical model of the bone and the second is the same anatomical model, but with modeled implant structure bulging from it. When the computer calculates the difference between these models - the resultant structure is the implant (Figs. 6, 7).

#### Final steps

**Fig. 8:**
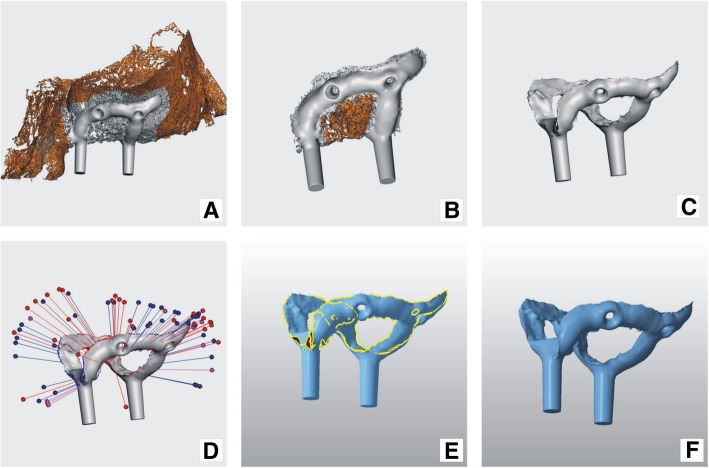
Implant model cleaning and repair: **a**) and **b**) unwanted shells selected (orange) for deletion in Meshmixer program; **c**) implant cleaned from unwanted snowflake like shells; **d**) implant model errors shown in Meshmixer program; **e**) implant model errors shown in NetFabb program; **f**) repaired implant in Netfabb program

Implant contains small residual artifacts that have the appearance of small snowflake-like structures left behind after the Boolean operation. These small objects appear because of inconsistencies accumulated in virtual models during earlier manipulations. Such structures have to be removed manually and errors corrected using the above-mentioned software (Figs. 7e, 8a and b).

#### Model repair

Model mesh repair is a very important part of the modeling process, from the start to the end. Initially, it is necessary to repair the virtual model of the jawbone before any modifications to the virtual models (before modeling) is performed. Further, it is necessary to repair models before and after each subtraction or addition operation when needed, because software cannot commence Boolean operations on meshes containing errors. Finally, designed implant models also need to be checked for errors and repaired if needed, before feeding the mesh file to the machine code generation program. There are many types of mesh errors that can occur in complex models of human anatomy—bones in particular. These include, but are not limited to, flipped triangles, holes, non-manifold edges, duplicate faces, and duplicate vertices. Usually, mesh repair is a multi-step process that corrects various types of errors.

There are various freely available programs available for mesh error repair. They include the following:

▪ Netfabb Basic (comprehensive list of errors repaired, automated scripts).

▪ MeshLab (no automated scripts, need manual selection for each type of errors to be corrected).

▪ 3Data expert lite (limited repair capabilities in free version).

▪ GOM Inspect V8 SR1 (some error repair capability).

▪ Meshmixer (automated error repair, several repair options, not sufficient even for program internal needs).

Netfabb contains pre-compiled repair scripts for correcting errors of different levels of complexity. This option facilitates user operations with mesh repair. Netfabb Private is delivers three pre-compiled repair scripts for mesh repair. These three scripts are of increasing comprehensiveness for error detection and correction(Table 2).

**Table 2:** Preinstalled repair scripts of Netfabb program

Simple repair	Default repair	Extended repair
• close trivial holes	• close trivial holes	• close trivial holes
• close all holes	• close all holes	• close all holes
• fix flipped triangles	• fix flipped triangles	• fix flipped triangles
	• stitch triangles	• stitch triangles
	• remove double triangles	• remove double triangles
	• remove tiny shells	• remove tiny shells
	• remove degenerated faces	• remove degenerated faces wrap part surfaces

The “default repair” script of the Netfabb program was used at all steps where it was necessary (Figs. 8d and e).

#### Printing

**Fig. 9:**
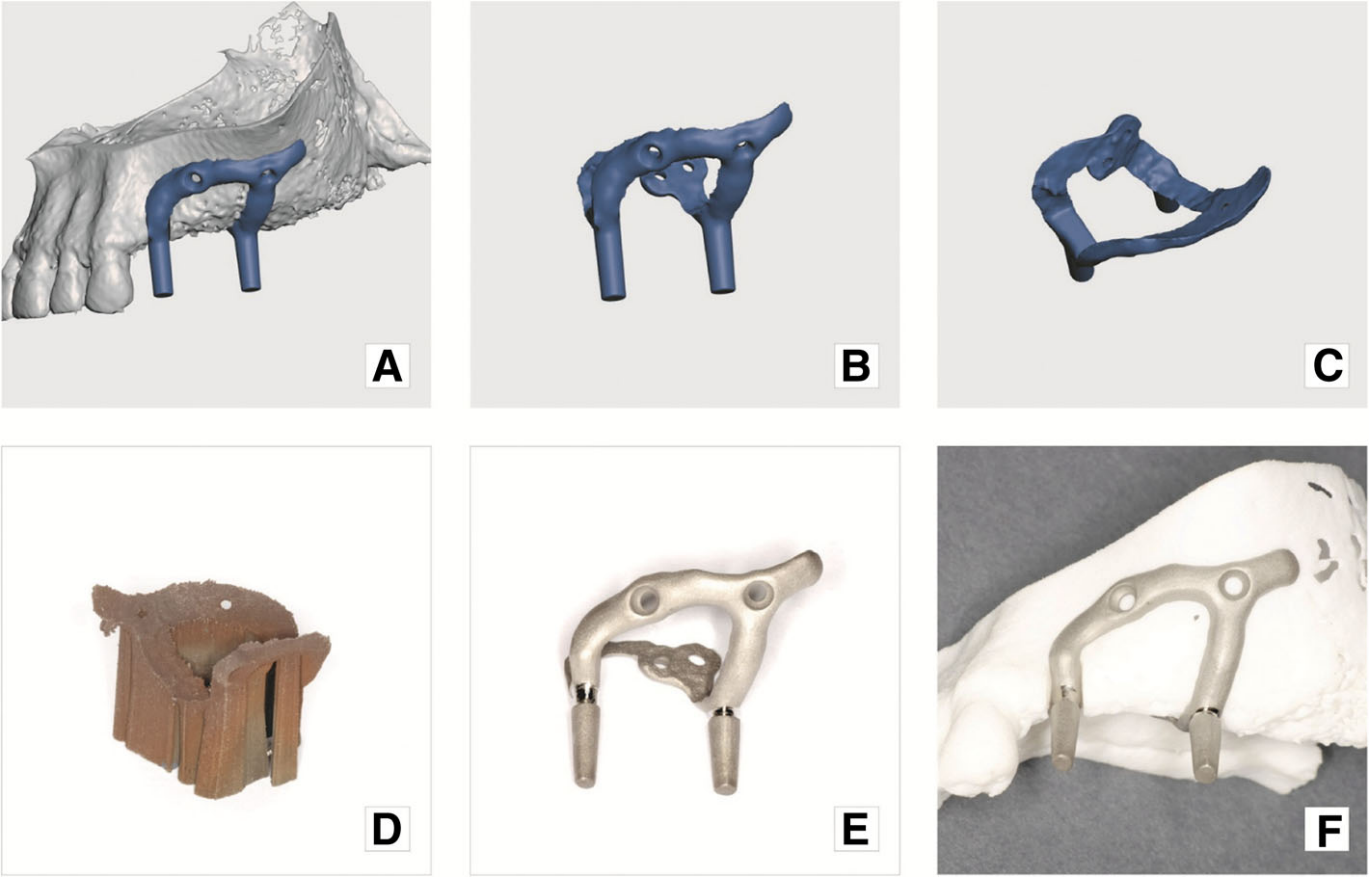
Final result: **a**) implant digital model superimposed on the jaw virtual model; **b**) and **c**) implant digital model in different projections; **d**) implant with printing supports, immediately after production; **e**) implant with supports removed and mechanical grinding and polishing completed; **f**) subperiosteal implant on the plastic model of maxilla

On completion of modeling, the implant design and jaw model STL files (Figs. 9a, b, c) were sent to a printing facility (Orthobaltic UAB, Lithuania).

The jawbone model, created earlier, was printed in polyamide using an SLS printer ESO P 396, in 60 mkm layers. The implant was produced using the EOS printer EOSINT M 280 by DMLS process in Ti64 alloy (100mkm layers), which is known to be biocompatible and exhibits the required mechanical properties. The implant was printed and then annealed in an argon environment. Supports were removed, and the implant was finished by a technician. The inner and outer surfaces of the implant were checked for voids or pimples. These were removed when encountered by abrasive instruments. Implant fit was checked against a plastic jaw model. In addition, implant outer surface was shaped to the desired profile with tungsten carbide cutters and blasted with aluminum oxide 150 mkm at 5 bar (Figs. 9d, e, f). Complete preparation of the implant for surgery includes more steps, which are metal surface treatments (polishing, blasting, etching, etc.), packaging and sterilization, but those are not in the scope of this article.

## Results

Patient CT data was successfully converted into a meshed surface (.stl) model of the maxilla using Slicer 3D, an open source software program.

Error corrections and design were completed using freely available Netfabb and Meshmixer programs from Autodesk Inc.. This article defines a workflow which allows for fast and high quality digital modeling of subperiosteal implants. The implant was produced in Ti64 allow using 3D printing DMLS process. Other aspects of subperiosteal implant use, such as implant surface treatment, surgical and prosthetic steps are out the scope of current article. Clinical results using 3D printed subperiosteal implants will be reported in future publications.

Legal background. Patient-specific (custom-made) implants are not required to be CE certified. According to Medical Device Directive (MDD) 93/42/EEC, “custom-made medical device - Any device specifically made in accordance with a duly qualified medical practitioner’s written prescription which gives, under his responsibility, specific design characteristics and is intended for the sole use of a particular patient”. Patient-specific (custom-made) implants must comply with the relevant essential requirements established in MDD as applicable to ensure their safety.

Our implants are registered in the State Health Care Accreditation Agency under the Ministry of Health of the Republic of Lithuania as medical devices that fully comply MDD and Lithuanian Medical Norm MN 4:2009 “Medical Devices Safety Technical Regulation”.

The software used for modeling of custom medical devices is also considered medical device. Computer programs which process data derived from patients usually considered to be Class IIb medical device. In European Union they are required to be CE certified. Neither of programs used in this publication are CE certified. According to authors knowledge (to the date 2019–04) there is only one medical device design software package which CE certified. It is 3-matic Medical by Materialise. Though, its cost is prohibitive to small companies.

## Conclusions

Custom dental implants have their niche in modern dental practice, serving patents with advanced maxillary or mandibular bone resorption. This type of medical device can be designed using freely available or inexpensive software tools from CT scans and produced by current 3D manufacturing technology to the required precision and fit. Numerous factors have to be taken into account by medical device designers as implant modeling quality is affected by the following:

1. CT scan data quality
2. Segmentation software algorithms and settings
3. modeling software output quality
4. Error repair software algorithms

Development of specialized software for this application would be advantageous for dental specialists, producers, and patients.

## Abbreviations

CAD/CAM: computer assisted design/computer assisted manufacturing
CoCr: cobalt and chromium alloy
CT: computed tomography, computer tomography
DICOM: Digital Imaging and Communications in Medicine (DICOM) is a standard for storing and transmitting medical images
DMLS: direct metal laser sintering
FDI: The FDI Dental Numbering System for Adult Teeth
SLS: selective laser sintering
STL: surface tessellation language; a file format
Ti64: titanium, aluminum 6%, vanadium 4% alloy
