# Publisher Correction to: 3D Printing in Medicine, volume 5

**DOI:** 10.1186/s41205-019-0051-1

**Published:** 2019-08-20

**Authors:** 3D Printing in Medicine

**Affiliations:** London, UK

**Publisher Correction to: 3D Printing in Medicine, volume 5**

**https://doi.org/10.1186/s41205-019-0046-y**

An error occurred during the publication of one article in *3D Printing in Medicine*. This article was published in volume 5 with a duplicate citation number.

In this correction article the old and new citation metadata are published in Table 1.

**Table 1:** Overview of incorrect and correct citation metadata

DOI	Incorrect citation number	Correct citation number
10.1186/s41205-019-0046-y	1	9

The original article has been updated. The publisher apologizes for the inconvenience caused to our authors and readers.

